# Irradiated mesenchymal stem cells support stemness maintenance of hepatocellular carcinoma stem cells through Wnt/β-catenin signaling pathway

**DOI:** 10.1186/s13578-020-00449-5

**Published:** 2020-08-03

**Authors:** Jing Hou, Naping Zhao, Pengxi Zhu, Jun Chang, Yan Du, Wei Shen

**Affiliations:** 1 GCP Office, Xinhua Hospital Affiliated to Shanghai Jiaotong University School of Medicine, 1665 Kongjiang Road, Shanghai, 200092 People’s Republic of China; 2 Department of Pharmacy, Changhai Hospital, Naval Military Medical University, 168 Changhai Road, Shanghai, 200433 China; 3 Department of Pharmacy, Naval Military Medical University, 800 Xiangyin Road, Shanghai, 200433 China

**Keywords:** Mesenchymal stem cells, Liver cancer stem cells, Tumor microenvironment, Wnt/β-catenin signaling pathway

## Abstract

**Background:**

Cancer stem cells are the main reason of relapse, metastasis and resistance to anti-cancer therapies of Hepatocellular carcinoma (HCC). Mesenchymal stem cells (MSCs) are an important part of the tumor microenvironment. MSCs have been demonstrated to be involved in drug resistance in tumor. How MSCs contribute to radiotherapy resistance of HCC is still indistinct.

**Methods:**

Flow cytometry analysis was performed to isolate CD133+ cells from HCC cell lines Huh7 and PLC. The stemness of Huh7-CD133 and PLC-CD133 those were co-cultured with IR-MSCs were investigated by Colony formation assay. Tumor formation in nude mice was used to explore the tumorigenicity of CD133+ cancer cells. The activating Wnt/β-catenin signaling pathway in CSCs were also detected by RT-PCR and Western blotting.

**Results:**

We report that irradiated MSCs (IR-MSCs) could increase the ratio of CD133^+^ cells in hepatocellular carcinoma cells. IR-MSCs could promote stemness maintenance of HCC stem cells. After co-cultured with IR-MSCs, liver cancer stem cells (CSCs) presented increased colony formation ability and tumor formation ability. We also found IR-MSCs promoted Wnt expression of CSCs. Reverse suppression experiment showed that when Wnt inhibitor was added into the culture medium, the effect of IR-MSCs on stemness maintenance was counteracted.

**Conclusions:**

These data showed that IR-MSCs could support stemness maintenance of CSCs by activating Wnt/β-catenin signaling pathway.

## Background

HCC is one of the most common malignant tumors in the world. It is easy to metastasize and relapse, and seriously endangers human life. High expression of multidrug resistance genes in liver cancer results in insensitive to chemotherapy. For patients with liver cancer who have lost their chance of surgery, radiation therapy has gradually become an important treatment for primary liver cancer. However, some patients are still not sensitive to radiotherapy, so enhance the radiotherapy sensitivity and effective treatment of liver cancer are particularly urgent and important.

In the study of tumor origin, CSCs have become a hotspot and attract more and more attention. With the discovery of subpopulations with the characteristics of CSCs in liver cancer cell lines, more and more evidence indicates that liver cancer stem cells are the main cause of liver cancer [1, 2].The characteristics of CSCs are very similar to those of normal stem cells, such as self-renew and multi-directional differentiation. And maybe only a small number of such cells control tumor growth [3]. Numerous studies confirm that tumor occurrence, development, metastasis and recurrence and chemotherapy resistance are closely related to CSCs [4, 5]. The insensitivity of liver CSCs to radiotherapy is also the main cause of insensitivity of HCC to radiotherapy. By using CD133 and other surface markers, the researchers isolated a population of stem cell-like tumor cells from liver cancer cell lines and liver cancer tissues that showed similar proliferation, self-renewal and differentiation capabilities to stem cells, also showed strong tumor formation ability in nude mice. The growth of liver CSCs is inseparable from the support of liver cancer microenvironment. How does the liver cancer microenvironment affect the stemness of CSCs?

With the development of molecular biology technology, it has been found that the tumor microenvironment plays an important role in tumor evolution, while MSCs are an important adult stem cells that constitutes the tumor microenvironment. MSCs have the characteristics of supporting hematopoiesis, immune regulation and multi-directional differentiation, and can also migrate to damaged tissues, chronic inflammatory reaction sites and repair damaged tissues [6]. Tumors have been described as non-healable lesions, and a large number of studies have shown that MSCs have tumor tropism. After MSCs homing to tumor tissue, how does it work on tumor cells? In some animal tumor models.

Exogenous MSCs can promote melanoma, colon cancer, multiple myeloma, lung cancer, and glioblastoma Progression of tumor [7–9]. How MSCs promote resistance of radiotherapy of CSCs need a further study. In this study, we isolated CSCs from HCC cell lines by using CD133 marker. We found that irradiated MSCs (IR-MSCs) could promote stemness maintenance of liver CSCs. After co-cultured with IR-MSCs, CSCs presented increased colony formation capability and tumor formation in nude mice.

Wnt/β-catenin signaling pathway is a highly conserved pathway in biological evolution, and is regulated by a series of small proteins inside and outside the cell. It has been known to play an important role in embryonic cell development, proliferation, transformation, cell adhesion, cell survival and apoptosis [10]. In recent years, with the gradual expansion of research on Wnt signaling pathway, it has been found that signaling molecules of Wnt pathway are involved in the process of tumors stem cell self-renewal regulation [11, 12].In normal breast cells, the expression of Wnt signaling pathway-related proteins is restricted, and its expression is increased in breast cancer cells, suggesting that Wnt signaling pathway is responsible for the maintenance of breast cancer stem cells [13]. Wnt signaling pathway also plays an important role in the self-renewal and differentiation of liver CSCs. In a further study, we found that IR-MSCs could activate Wnt signaling pathway. In addition, Wnt inhibitor counteracted the effect of IR-MSCs on stemness maintenance of liver CSCs. Thus, the data showed that IR-MSCs supported maintaining stemness of CSCs through Wnt/β-catenin signaling pathway.

## Results

### IR-MSCs increased proportion of CD133 positive cells in hepatocellular carcinoma cells

**Fig. 1:**
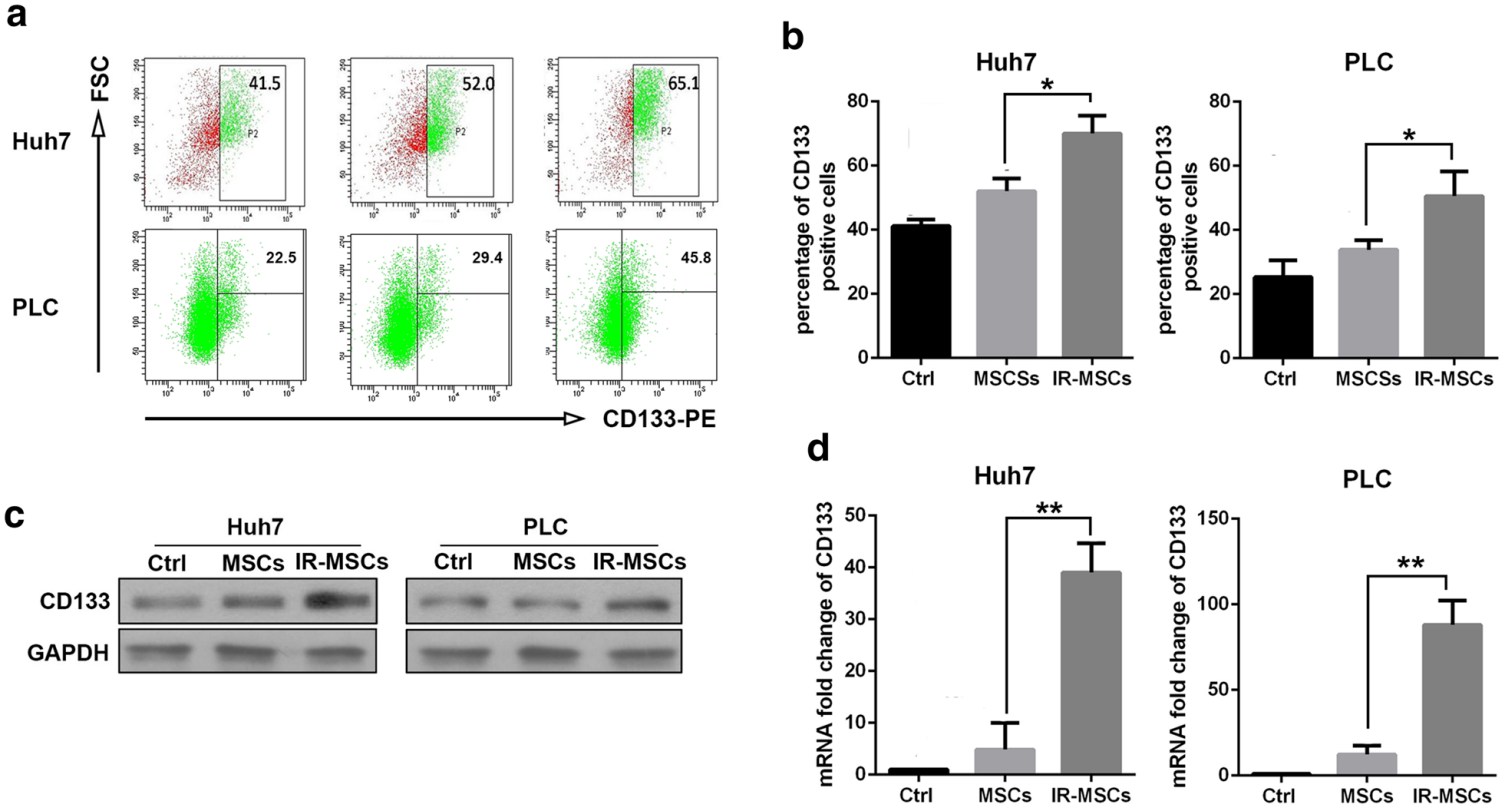
IR-MSCs increased proportion of CD133 positive cells in hepatocellular carcinoma cells. CD133 detection in Huh7 and PLC after co-culture with MSCs and IR-MSCs for 7 days. **a** Detection of CD133 positive cells proportion in HCC cell lines Huh7 and PLC by flow cytometry. **b** Quantification of panel A. **c** Protein expression of CD133 detected by western blotting assay. **d** mRNA expression of CD133 detected by RT-PCR. IR-MSCs, irradiated MSCs

Umbilical cord mesenchymal stem cells were presents from Dr. Shi in the Institute of health sciences. MSCs were irradiated by 6 Gy and then recovered for 12 h. Hepatocellular carcinoma cells Huh7 and PLC were co-cultured with IR-MSCs or control MSCs for 7 days. Then CD133 was detected on the surface of Huh7 and PLC by flowcytometry. As shown in Fig. 1a, b, after co-culture with MSCs, the proportion of CD133 positive cells did not change significantly. Whereas, compared with MSCs group, the proportion of CD133 positive cells was increased significantly after co-culture with IR-MSCs. In addition, western blotting and RT-PCR results showed that IR-MSCs promoted expression of CD133 in Huh7 and PLC both at mRNA level and protein level (Fig. 1c, d).

### IR-MSCs promoted stemness maintenance of CD133 positive cells

**Fig. 2:**
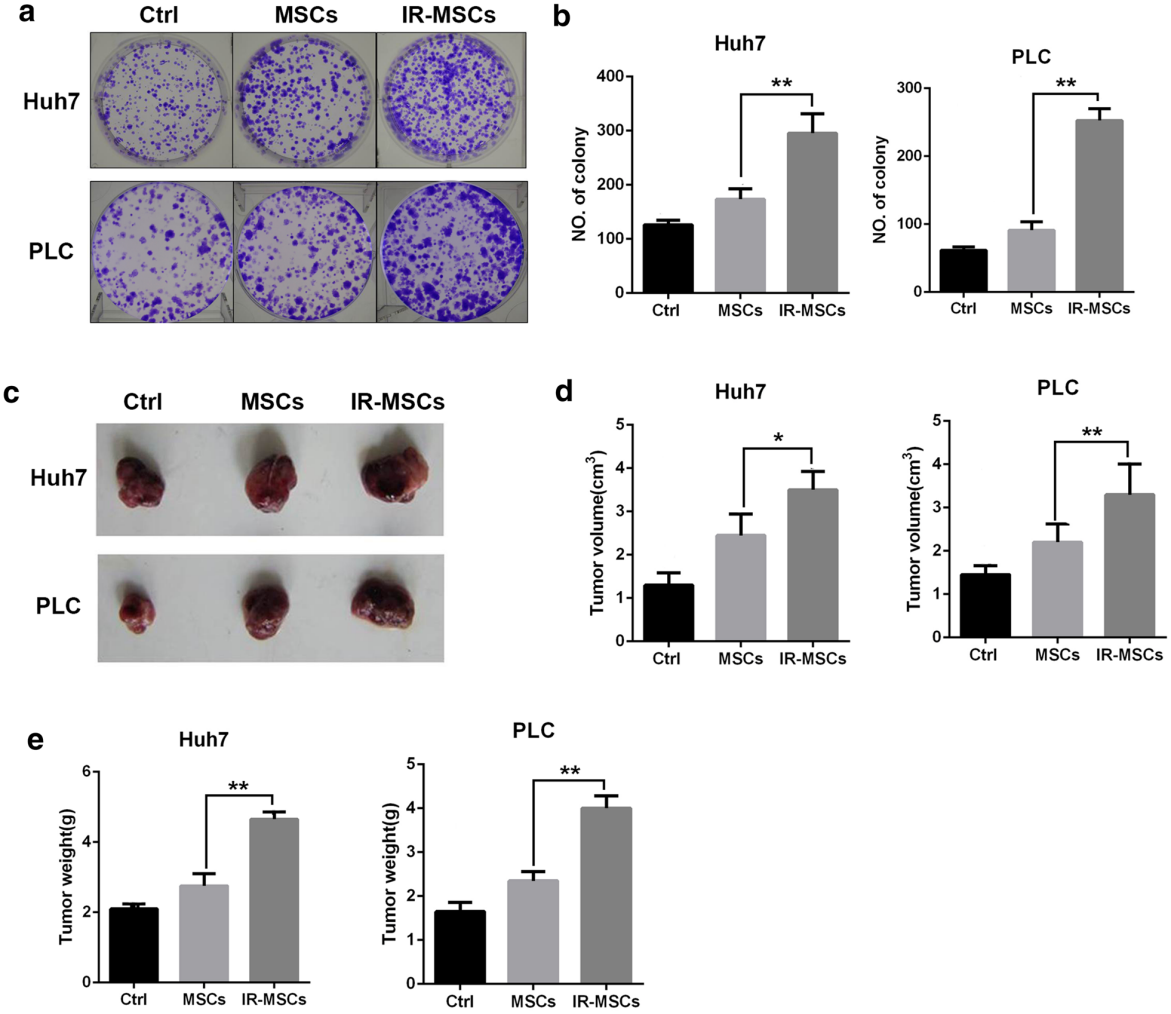
IR-MSCs promoted stemness maintenance of CD133 positive cells. Colony formation and tumorigenesis ability in vivo of CSCs isolated from Huh7 and PLC after co-cultured with MSCs and IR-MSCs. **a** Colony formation of CD133+ Huh7 and CD133+ PLC. **b** Quantification of panel **a**. **c** Tumorigenesis of CD133+ Huh7 and CD133+ PLC in nude mice. **c**–**e** Tumor volume and tumor weight were measured. IR-MSCs, irradiated MSCs

To investigate the effect of IR-MSCs on stemness maintenance of hepatocellular carcinoma cells, we isolated CD133^+^ cells from Huh7 and PLC as tumor stem cells, then CD133^+^ cells were co-cultured with IR-MSCs. 7 days later, Huh7-CD133 and PLC-CD133 were plated into 6 well plate as 500 cells per well. Then colony was stained by crystal violet and counted 7 days after culture. In addition, we detected tumorigenesis of tumor stem cells. 1 × 10^6^ Huh7-CD133 and PLC-CD133 were injected into nude mice subcutaneously after co-culture with IR-MSCs for 7 days. 4 weeks later, tumor volume and weight were tested in different groups. As shown in Fig. 2c, IR-MSCs could promoted tumor formation of tumor stem cells at a great degree. As shown in Fig. 2d, e, tumor volume and tumor weight was increased after co-cultured with IR-MSCs.

### IR-MSCs promoted Wnt signaling pathway

**Fig. 3:**
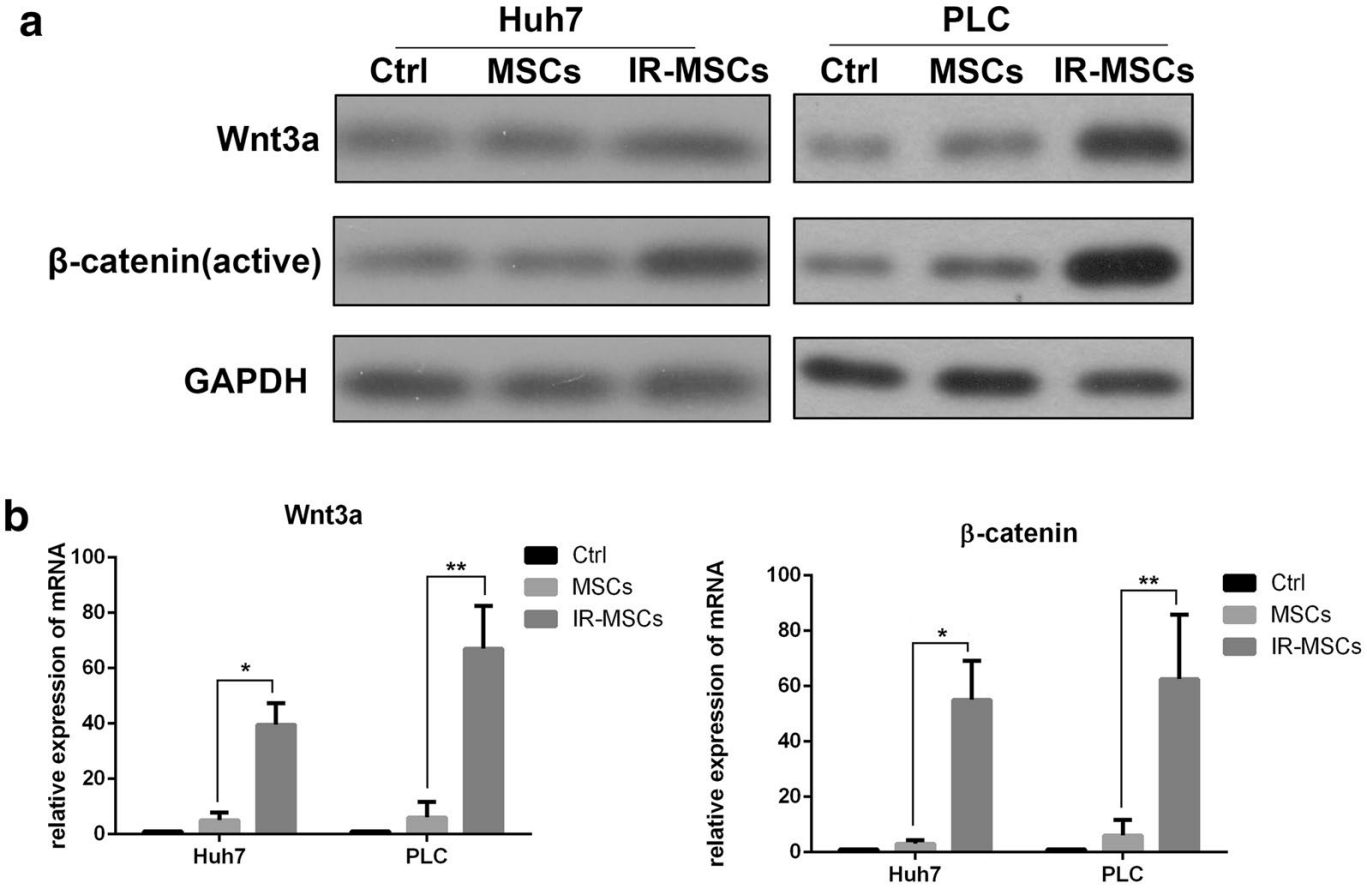
IR-MSCs promoted Wnt signaling pathway. **a** Wnt3a and β-catenin expression in CD133+ Huh7 and CD133+ PLC after co-cultured with MSCs and IR-MSCs. **b** mRNA expression of Wnt3a and β-catenin in CD133+ Huh7 and CD133+ PLC

Wnt/β-catenin signaling pathway play a key role in the process of tumors stem cell self-renewal regulation [11]. Thus, we detected Wnt and β-catenin expression in tumor stem cells. We detected the gene expression of Wnt family members by Real-Time PCR. The data showed the expression of Wnt3a in IR-MSCs group was higher than MSCs group(Additional file 1: Fig. S1).As shown in Fig. 3a, after co-culture with IR-MSCs, both Wnt and β-catenin were increased in Huh7-CD133 and PLC-CD133. Data in Fig. 3b also showed the same result at mRNA level.

### Inhibition of Wnt signaling pathway could suppress the function of IR-MSCs

**Fig. 4:**
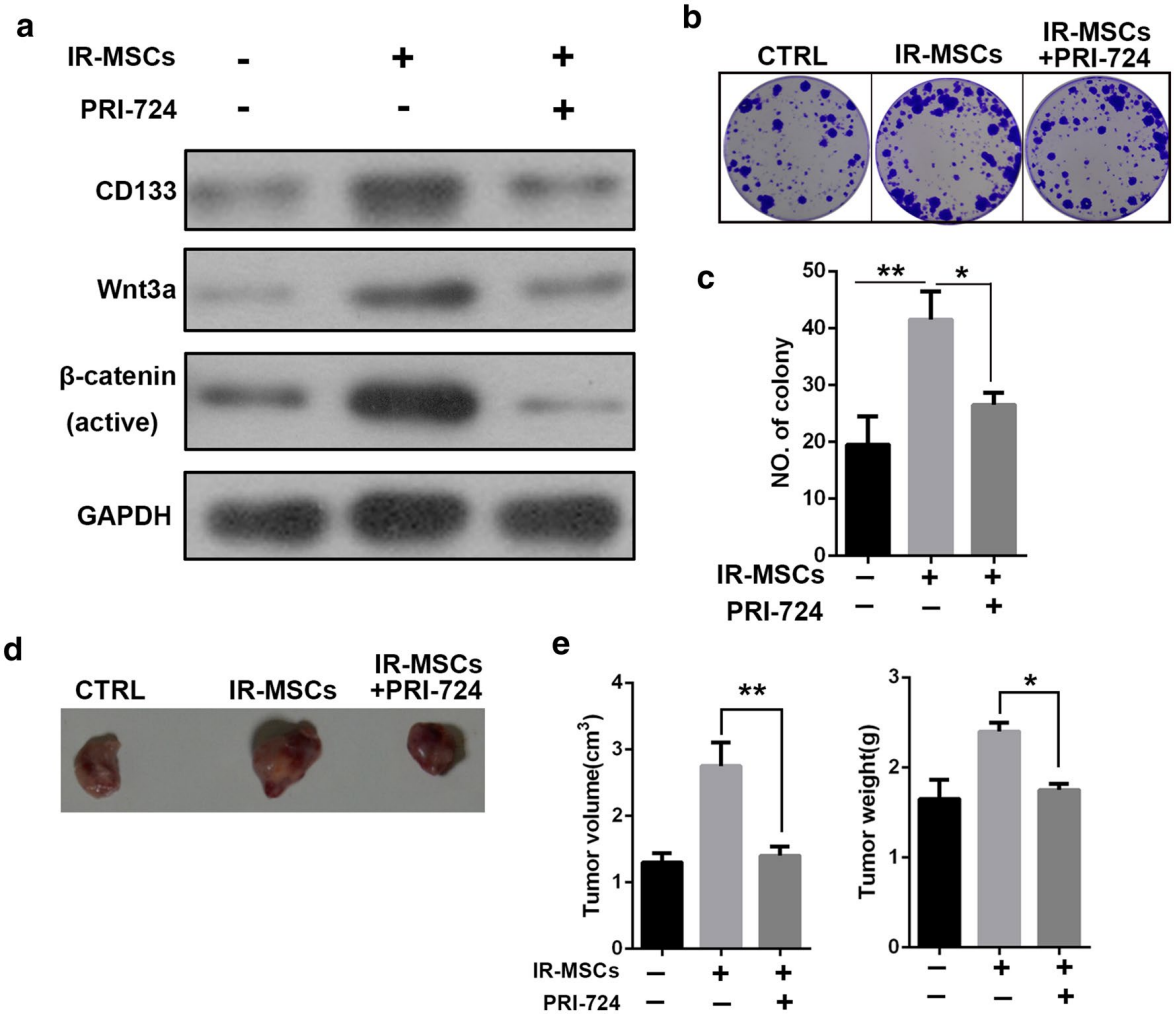
Inhibition of Wnt signaling pathway could suppress the function of IR-MSCs. CD133+ Huh7 was pretreated by PRI-724 and co-cultured with MSCs and IR-MSCs for 7 days. **a** Expression of CD133, Wnt3a and β-catenin in CD133+ Huh7. **b** Colony formation of CD133+ Huh7. **c** Quantification of panel B. **d** Tumorigenesis of CD133+ Huh7 in nude mice. **e** Tumor volume and weight were measured

To verify the role of Wnt/β-catenin signaling pathway in stemness maintenance of tumor stem cells, we used inhibitor of Wnt signal, PRI-724. Huh7-CD133 was treated by PRI-724(30 nM)for 48 h, then co-cultured with IR-MSCs. We found that PRI-724 could reverse the enhanced effect of IR-MSCs on CD133 expression, colony formation and tumorigenesis in nude mice of tumor stem cells (Fig. 4).

## Discussion

Radiotherapy resistance of HCC is becoming more and more common which affects therapy of HCC patients. While, the mechanism of HCC radiotherapy resistance is not clear. Increasing evidence indicated that CSCs were the main reason of radiotherapy resistance. CSCs describe a class of stem cells capable of self-renewal and multi-differentiation [14–17] which can maintain the proliferation and growth of tumors and evade endogenous and exogenous regulation of the stability of their internal environment. These characteristics of what is known as “stemness” allow for the growth of the primary cancer tumor as well as the development of new tumors, metastasis and recurrence of tumors [18, 19]. CSCs can survive while normal cancer cells die under radiotherapy. CD133 has been reported to be a marker of CSCs in many tumors, such as breast, prostate, colon, glioma, liver, lung, ovary cancer [20]. Numerous factors, including the central role of the tumor microenvironment, have been hypothesized to contribute to CSC treatment resistance [21].

Mesenchymal stem cells are a group of adult stem cells which can migrate to tissue injury sites and tumor sites. MSCs have been reported to be very important in tissue injury repair and tumor formation [6]. In former study, we found that MSCs could maintain their stemness after radiotherapy through autophagy activation [22]. In this study, we isolated CSCs from HCC cell lines Huh7 and PLC by using CD133 positive screening and co-cultured MSCs with CSCs. We found that compared with normal MSCs, irradiated MSCs could support stemness maintenance of cancer stem cells. Expression of stem cell markers, ability of colony formation and tumorigenesis in vivo were increased after co-cultured with IR-MSCs.

Wnt/β-catenin signaling pathway has been known to play an important role in embryonic cell development, proliferation, transformation, cell adhesion, cell survival and apoptosis [10]. In recent years, with the gradual expansion of research on Wnt signaling pathway, it has been found that signaling molecules of Wnt pathway are involved in the process of tumor stem cell self-renewal regulation [11]. We found that IR-MSCs could upregulate Wnt and β-catenin expression. In order to verify the role of Wnt signaling pathway in stemness maintenance of CSCs, we used Wnt signal inhibitor PRI-724 to pre-treat CSCs to block Wnt signaling pathway in CSCs. Data showed that the effect of stemness support of IR-MSCs was inhibited after PRI-724 pretreatment. Thus, IR-MSCs may support stemness maintenance of CSCs through activating Wnt/β-catenin expression.

However, what happens to MSCs after irradiation also needs our thinking. Recently, some studies found non-coding RNAs can active Wnt signaling in cancer stem cells.

In mammary stem cells, miR-142 recruits the APC mRNA for degradation and increases Wnt signaling [23]. The long non-coding RNA lncTCF7, which is expressed in liver CSCs, was found to activate Wnt signaling is associated with an increased self-renewal capacity of liver CSCs [24]. The other studies showed multiple cytokines exist in tumor microenvironment [25, 26], such as transforming growth factor (TGF-), epidermal growth factor (EGF), Insulin-like growth factor (IGF), vascular endothelial growth factor (VEGF) drive cross-talk between tumor cells and stromal cells. What factor is secreted by IR-MSCs to affect the stemness maintenance of CSCs still needs a further study.

## Conclusions

Our data showed that IR-MSCs could support stemness maintenance of CSCs by activating Wnt/β-catenin signaling pathway.

## Materials and methods

### Irradiation

MSCs were plated into T25 plates. MSCs were irradiated with ELEKTA Synergy Linear Accelerator (Cravoley, UK) at 6 Gy (a dose rate of 350 cGy/min) over an appropriate field size when they were 80% confluent. Irradiated cells were allowed to recover for 12 h.

### Isolation of CD133+ populations with flow cytometry

To isolate CD133+ cells from HCC cell lines Huh7 and PLC, both cells were suspended as single cell suspension, then labeled with PE-conjugated anti-CD133 antibody for 30 min, then CD133+ cells were isolated apart from CD133- cells by flow cytometry. They were named as Huh7-CD133 and PLC-CD133, respectively.

### Co-culture assay

Transwell assay was used to perform co-culture assay. Huh7 and PLC were plated in 6-well plate at a density of 1Χ10^5^, MSCs or IR-MSCs were seeded in transwell insert(0.4 μm pore), after co-culture for seven days, Huh7 and PLC were used for other experiments.

### CD133 detection with flow cytometry

HCC cell lines Huh7 and PLC were co-cultured with IR-MSCs for 7 days, then the expression of CD133 was measured by flow cytometry. Cells were labeled with PE-conjugated anti-CD133 antibody, the percentage of CD133 + cancer cells was measured by MACSQuant Analyzer10 flow cytometric system (Miltenyi Biotech, Germany).

### Colony formation assay

Huh7-CD133 and PLC-CD133 were co-cultured with IR-MSCs for 7 days, then were seeded into 6 well plate as 500 cells per well. 7 days later, cells were fixed and stained by 0.1% crystal violet solution. The number of colonies larger than 2 mm in diameter was counted.

### Tumor formation in nude mice

Six-week-old male athymic BALB/c nu/nu mice were obtained from Shanghai Experimental Animal Center, Chinese Academy of Science. Mice were maintained under a pathogen-free condition and treated in accordance with the institutional animal welfare guidelines. For tumorigenicity assay, Huh7-CD133 and PLC-CD133 were co-cultured with IR-MSCs for 7 days. 1 × 10^6^ cells in 100 μL were injected subcutaneously to the left back of mice. Mice were sacrificed 4 weeks after injection. Tumors were collected and volume and weight of tumors were calculated.

### RT-PCR

Total RNA was abstracted by Trizol assay and reverse-transcripted to cDNA by bestar qPCR RT kit. mRNA expression was detected by RT-PCR by using bestar real-time PCR master mix with an ABI Prism 7300 system. The primers are as follows: CD133, sense, AGTCGGAAACTGGCAGATAGC, antisense, GGTAGTGTTGTACTGGGCCAAT; Wnt3a, sense, AGCTACCCGATCTGGTGGTC, antisense, CAAACTCGATGTCCTCGCTAC; β-catenin, sense, AGCTTCCAGACACGCTATCAT, antisense, CGGTACAACGAGCTGTTTCTAC; GAPDH, sense, GGAGCGAGATCCCTCCAAAAT, antisense, GGCTGTTGTCATACTTCTCATGG. The primers for genes of Wnt family (Additional file 2: Table S1).

### Western blotting

Total protein was acquired by RIPA and quantified by BCA assay. Proteins of the same mass were tested for expression of CD133 (64326, 1:1000,Cell signal technology) and Wnt3a (ab28472, 1:1000, abcam), β-catenin (ab6302, 1:1000, abcam), GAPDH(ab8245, 1:5000, abcam)by SDS-PAGE, GAPDH was used as control. The assay was performed according to protocols described before [27].

### Statistical analysis

All the experiments were performed 4 times. Student T-test was done to analysis the difference between different groups. *P < 0.05 and **P < 0.01 represent a statistical difference. Data were expressed as the mean ± standard deviation (SD).

## Supplementary information

**Additional file 1: Figure S1.** Gene expression of Wnt family. Huh7 and PLC were co-cultured with ctrl MSCs or IR-MSCs for seven days, then mRNA was abstracted and RT-PCR assay was performed to detect expression of Wnt1,Wnt2, Wnt3, Wnt3a, Wnt4, Wnt5a, Wnt6, Wnt7b, Wnt10b,and Wnt11.

**Additional file 2: Table S1.** RT-PCR Primers for genes of Wnt family.
