# Supplementary figures and images for: Irradiated mesenchymal stem cells support stemness maintenance of hepatocellular carcinoma stem cells through Wnt/β-catenin signaling pathway
Source: Cell Biosci. 2020 Aug 3;10:93. doi: 10.1186/s13578-020-00449-5 (PMC7398068; doi:10.1186/s13578-020-00449-5)

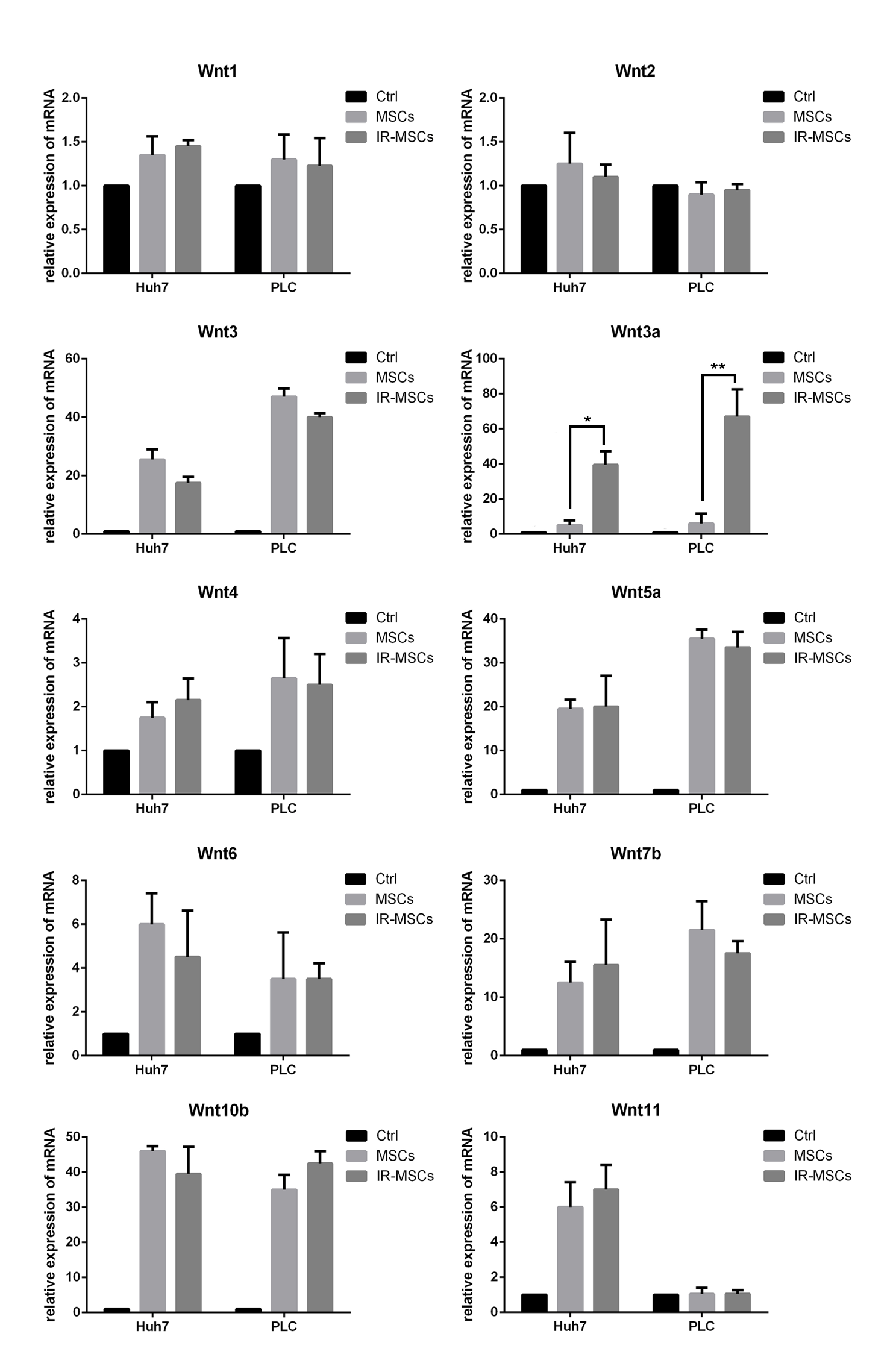

Supplement: Supplementary file 1 — Additional file 1: Figure S1. Gene expression of Wnt family. Huh7 and PLC were co-cultured with ctrl MSCs or IR-MSCs for seven days, then mRNA was abstracted and RT-PCR assay was performed to detect expression of Wnt1,Wnt2, Wnt3, Wnt3a, Wnt4, Wnt5a, Wnt6, Wnt7b, Wnt10b,and Wnt11. [file 13578_2020_449_MOESM1_ESM.tif]
