# Supplementary material for: Irradiated mesenchymal stem cells support stemness maintenance of hepatocellular carcinoma stem cells through Wnt/β-catenin signaling pathway
Source: Cell Biosci. 2020 Aug 3;10:93. doi: 10.1186/s13578-020-00449-5 (PMC7398068; doi:10.1186/s13578-020-00449-5)
Supplement: Supplementary file 2 — Additional file 2: Table S1. RT-PCR Primers for genes of Wnt family. [file 13578_2020_449_MOESM2_ESM.pdf]

TableS1. RT-PCR Primers for genes of Wnt family.

Gene	Primer Sequence (5' -> 3')	
	Forward Primer	Reverse Primer
Wnt1	CGATGGTGGGGTATTGTGAAC	CCGGATTTTGGCGTATCAGAC
Wnt2	CCGAGGTCAACTCTTCATGGT	CCTGGCACATTATCGCACAT
Wnt3	CTCGCTGGCTACCCAATTTG	AGGCTGTCATCTATGGTGGTG
Wnt4	AGGAGGAGACGTGCGAGAAA	CGAGTCCATGACTTCCAGGT
Wnt5a	ATTCTTGGTGGTCGCTAGGTA	CGCCTTCTCCGATGTACTGC
Wnt6	GGCAGCCCCTTGGTTATGG	CTCAGCCTGGCACAACTCG
Wnt7b	CACAGAAACTTTCGCAAGTGG	GTACTGGCACTCGTTGATGC
Wnt10b	CATCCAGGCACGAATGCGA	CGGTTGTGGGTATCAATGAAGA
Wnt11	GGAGTCGGCCTTCGTGTATG	GCCCGTAGCTGAGGTTGTC
